# Fasting plasma glucose level and the risk of open angle glaucoma: Nationwide population-based cohort study in Korea

**DOI:** 10.1371/journal.pone.0239529

**Published:** 2020-09-23

**Authors:** Jin A. Choi, Yong-Moon Park, Kyungdo Han, Jiyoung Lee, Jae-Seung Yun, Seung-Hyun Ko

**Affiliations:** 1 Department of Ophthalmology and Visual Science, St. Vincent’s Hospital, College of Medicine, The Catholic University of Korea, Seoul, Korea; 2 Epidemiology Branch, National Institute of Environmental Health Sciences, National Institutes of Health, Research Triangle Park, North Carolina, United States of America; 3 Department of Statistics and Actuarial Science, Soongsil University, Seoul, Korea; 4 Division of Endocrinology and Metabolism, Department of Internal Medicine, St. Vincent’s Hospital, College of Medicine, The Catholic University of Korea, Seoul, Korea; University of Melbourne, AUSTRALIA

## Abstract

**Background:**

The level of fasting plasma glucose (FPG) is positively associated with intraocular pressure. Diabetes causes early structural changes of retina, especially on ganglion cell layer. In this regard, the FPG level itself may also show an independent association with open angle glaucoma (OAG) development in general population. Herein, we investigate the association of the FPG level with the incidence of OAG.

**Methods:**

Using nationally representative data from the Korean National Health Insurance System, 374,376 subjects ≥40 years of age without OAG who underwent health examinations from 2009 to 2013 were enrolled and followed to the end of 2014. Incident cases of OAG using the International Classification of Diseases 10 codes and medication information were analyzed based on the levels of FPG.

**Results:**

Subjects with the highest FPG level (≥160 mg/dL), compared with the lowest level (<80 mg/dL), showed a higher hazard ratio (HR) [2.189; 95% confidence interval (CI): 1.779–2.695; *P* for trend < 0.001] for OAG after adjustments for age and sex. This result persisted after further adjustments for the presence of type 2 diabetes, current smoking, drinking, and exercise habits, diastolic blood pressure, body mass index (BMI), and total cholesterol level (HR: 1.588; 95% CI: 1.268–1.989; *P* for trend < 0.001). The positive association between the FPG level and the incidence of OAG was distinct in subjects 40–64 years of age, those with a BMI <25 kg/m^2^, and those without hypertension (HR 2.022; 95% CI: 1.494–2.736; *P* for trend <0.001: HR 1.817; 95% CI: 1.372–2.407; *P* for trend < 0.001: HR 1.706; 95% CI: 1.180–2.467; *P* for trend <0.001, respectively).

**Conclusions:**

This nationwide population-based cohort study showed that the fasting glucose was associated with an increased risk of OAG. These findings suggest that subjects with high FPG levels require special attention when screening for glaucoma.

## Introduction

Type 2 diabetes mellitus (T2D) is a global pandemic, from industrialized nations to the emerging economies of Asia, Latin America, and Africa [1–3]. The public health burden of T2D is largely attributed to uncontrolled plasma glucose, which increases the likelihood of both macrovascular and microvascular complications. Diabetic retinopathy is one of the most well-known complications of T2D. However, T2D also accompanies many ocular complications such as glaucoma, corneal complications, optic disc abnormalities, and cataract [4], and glaucoma is potentially vision-threatening among the ocular complications associated with T2D [5].

The pathogenesis of glaucoma, in which selective loss of retinal ganglion cells occurs, includes a vascular component as well as a mechanical component [6]. T2D and open-angle glaucoma (OAG) share pathophysiologies such as microvascular endothelial dysfunction and impaired blood flow autoregulation [7]. Defects in the retinal nerve fiber layer, one of the pathognomonic findings in glaucomatous optic neuropathy, are frequently detected in subjects with T2D [6, 8].

Advanced imaging techniques offer an excellent opportunity to enhance our understanding of the early pathophysiological pathways of T2D and OAG. Recent studies have suggested that inner retinal thinning occurs before the development of diabetic retinopathy [9, 10]. Even in prediabetic stages, characterized by impaired fasting glucose and high insulin resistance, early vascular changes in the retina may occur [11, 12].

The fasting plasma glucose (FPG) level is used to diagnose T2D and prediabetic conditions, and it is a key indicator of future T2D and cardiovascular diseases [13]. FPG levels generally increase with advancing age [14], which is one of the cardinal risk factor of OAG. High FPG leads to high glucose levels in the aqueous humor, which can increase fibronectin in trabecular meshwork cells, resulting in increased intraocular pressure (IOP) [15]. Not only the presence of T2D but the level of FPG are associated with increased IOP [16–18]. Recent meta-analyses showed that the pooled average increase in IOP associated with a 10 mg/dL increase in the FPG level was 0.09 mmHg [17].

Considering the positive relationship between the level of FPG and IOP and the early changes of retinal ganglion cells in diabetes [16–18], the FPG level itself may also show an independent association with OAG development. In a cross-sectional study in the United States using 2005–2008 National Health and Nutrition Examination Survey, higher levels of FPG, fasting insulin, HbA1c, and homeostatic model assessment of insulin resistance (HOMA-IR) were associated with high prevalence of OAG in subjects with good glycemic control [18]. However, in another population-based cohort study in an adult Latino population, the relationship between random blood glucose level and the risk of OAG was not significant, whereas a significant association was noted between HbA1c with glaucoma, but this association was not independent from diabetes [19]. Despite the possible clinical significance, an association between the FPG level and OAG development has not been reported in large population-based cohort studies. To understand the possible association of the FPG level with OAG incidence better, we examined data from a nationally representative cohort of adults ≥ 40 years of age in the Republic of Korea.

## Materials and methods

## Database

In the Republic of Korea, the only health insurer is the National Health Insurance Service (NHIS), which is managed by the government and subscribed to by approximately 97% of the Korean population, either as an employee or, since 1989, as a community member [20]. The NHIS in the Republic of Korea manages the National Health Insurance Sharing Service, which oversees a comprehensive health-related database. This database is available to researchers for use in policy or academic efforts. The NHIS database in Korea includes detailed information on each registrant regarding demographics, outpatient and inpatient medical use, and claims for prescription drugs and procedures performed. Those enrolled in the health insurance service are recommended to undergo health check-ups at least biennially. Serum glucose levels were measured after an overnight 12-hour fast. FPG levels were categorized as <80, 80–99, 100–125, 126–139, 140–159, or ≥160 mg/dL. In all primary analyses, the FPG category < 80 mg/dL (< 5.0 mmol/L) was used as the reference group. The detailed cohort profile has been published elsewhere [21].

### Study sample

In the present study, we included 521,993 adults who underwent a health examination between January 1, 2009 and December 31, 2013. Of these subjects, we excluded subjects < 40 years of age (n = 140,473) and those who developed OAG from 2009 to 2013 (n = 4,445). Among the remaining 387,075 adults, subjects with missing values were also excluded (n = 12,699). Finally, 374,376 subjects were included in this study. The Institutional Review Board of St. Vincent’s Hospital, Catholic University in Seoul, Republic of Korea approved the protocols of the present study and also waived the need for informed consent (no. VC19ZESI0007). This study adhered to the tenets of the Declaration of Helsinki.

### Follow-up and definitions of parameters

Participants who enrolled between 2009 and 2013 were followed up from the date of the health examination to the date of OAG diagnosis or December 31, 2014. We defined patients with OAG using an OAG code (Korean Classification of Disease diagnostic code H401, corresponding to International Classification of Disease (ICD)-10 diagnostic code H40.1), in accordance with the former definition of OAG in published literature [21–24], comprising the following disorders: low-tension glaucoma (H4010), pigmentary glaucoma (H4011), capsular glaucoma with pseudoexfoliation of lens (H4012), residual stage of open-angle glaucoma (H4013), and unspecified primary open-angle glaucoma (H4019). We defined OAG by the diagnostic code, confirmed at more than one visit to an ophthalmologist, and a prescription for anti-glaucoma medication, in accordance with other published data [22, 23].

All the assessment for the main exposure (FPG measurement and diabetes status) and potential confounders were made at the time of enrollment using the results of the National Health Examination Program. A subject was defined as having T2D if prescribed oral hypoglycemic agents or insulin in addition to having an ICD-10 diagnostic code of E11, E12, E13, or E14. Regarding hypoglycemic medications, oral hypoglycemic agents (including metformin, sulfonylureas, meglitinides, thiazolidinediones, alpha-glucosidase inhibitors, dipeptidyl peptidase-4 inhibitors, or a combination of these different classes of hypoglycemic agents) and insulin use were included [20].

BMI was calculated as the subject’s weight (kg) divided by height squared (m). Information on current smoking (never smoker, former smoker, or current smoker), alcohol consumption (never drinker; moderate drinker, ≤ 1 drink per day; or heavy drinker, > 1 drink per day), and physical activity (no exercise; moderate exercise, 1–2 times per week; or regular exercise, ≥ 3 times per week) was obtained using a standard questionnaire during the health examination [25].

Hypertension was defined as a claim ICD-10 code of I10–I13 or I15 and a prescription for anti-hypertensive agents or a systolic or diastolic blood pressure (BP) ≥ 140 or ≥ 90 mmHg, respectively. Subjects with a systolic or diastolic BP ≥ 140 or ≥ 90 mmHg, respectively, and no prior exposure to anti-hypertensive agents were also investigated separately. Dyslipidemia was defined by a claim ICD-10 code of E78 and a prescription for lipid-lowering agents or a total cholesterol level ≥ 240 mg/dL.

### Statistical analyses

The characteristics of the study population based on the presence of T2D and OAG were analyzed using descriptive statistics. χ^2^ tests and independent *t-*tests were used for categorical and continuous variable comparisons, respectively. Person-time was calculated from the age at enrollment until the age of OAG diagnosis or until death, or last follow-up, whichever occurred first. Cox proportional hazards regression analysis was performed to compare the risk of OAG development among the groups stratified by the FPG level. The proportional-hazards assumption was evaluated using the Schoenfeld residuals test with the logarithm of the cumulative hazards function based on Kaplan–Meier estimates for the groups stratified by the FPG level. We assessed potential effect modification through stratified analyses which were performed to calculate the hazard ratios (HRs) according to age group (40–64 and ≥ 65 years), BMI (< 25 and ≥ 25 kg/m^2^), and BP (without hypertension, with hypertension without prior exposure to anti-hypertensive medication, and with hypertension and exposure to antihypertensive medication). The cumulative OAG incidence for each group was plotted using Kaplan–Meier curves according to the FPG category. Statistical software (version 9.4; SAS Institute, Cary, NC, USA) was used for all statistical analyses, and all statistical tests were two-tailed with a significance level set at *P* < 0.05.

## Results

Table 1 lists the characteristics of the study population according to the presence of T2D and OAG. Subjects with T2D were more likely to be male, older, and hypertensive and to have significantly higher systolic and diastolic BP, BMI, TG and FPG level and a significantly lower total cholesterol, HDL and LDL level, compared with those without T2D (all, *P* < 0.001). The patients with OAG were more likely to be male, older, and hypertensive and to have higher systolic/diastolic BP and FPG level compared with those without OAG (all, *P* < 0.001).

**Table 1:** Baseline characteristics of the study population according to the presence of type 2 diabetes and open-angle glaucoma.

	T2D		*P-*value	OAG		*P*-value
	No	Yes		No	Yes	
n	343376	31000		371360	3016	
**Age, years**	52.9 ± 10.9	60.9 ± 10.5	< 0.001	53.5 ± 11.1	61.5 ± 11.0	< 0.001
**Sex, male, %**	162113 (47.21)	16950 (54.68)	< 0.001	177506 (47.8)	1557 (51.62)	< 0.001
**Hypertension, %**	107926 (31.43)	21185 (68.34)	< 0.001	127521 (34.34)	1590 (52.72)	< 0.001
**Dyslipidemia, %**	73200 (21.32)	15913 (51.33)	< 0.001	88086 (23.72)	1027 (34.05)	< 0.001
**Smoker, %**						
Non	220006 (64.07)	18676 (60.25)	< 0.001	236685 (63.73)	1997 (66.21)	< 0.001
Ex	50140 (14.6)	5701 (18.39)		55326 (14.9)	515 (17.08)	
Current	73230 (21.33)	6623 (21.36)		79349 (21.37)	504 (16.71)	
**Drinker (>1 drink per day), %**	146559 (42.68)	10966 (35.37)	< 0.001	156477 (42.14)	1048 (34.75)	< 0.001
**Regular exercise, %**	145453 (42.36)	12748 (41.12)	< 0.001	156912 (42.25)	1289 (42.74)	0.591
**Glucose, mg/dL**	96.3 ± 17.9	142.5 ± 51.7	< 0.001	100.1 ± 26.0	106.6 ± 33.6	< 0.001
**Systolic BP, mmHg**	123.4 ± 15.5	128.8 ± 15.8	< 0.001	123.8 ± 15.6	127.3 ± 15.7	< 0.001
**Diastolic BP, mmHg**	76.8 ± 10.3	78.2 ± 10	< 0.001	76.9 ± 10.3	77.9 ± 10.3	< 0.001
**BMI, kg/m**^**2**^	23.9 ± 6.2	25 ± 3.3	< 0.001	24.0 ± 6.0	24.1 ± 3.0	0.401
**Total cholesterol, mg/dL**	199.7 ± 41	189.7 ± 47.1	< 0.001	198.9 ± 41.7	198.5 ± 39.4	0.595
**HDL cholesterol, mg/mL**	56.0 ± 28.5	51.3 ± 27.4	< 0.001	55.6 ± 28.5	54.9 ± 28.7	0.173
**LDL cholesterol, mg/mL**	119.1 ± 66.4	107.6 ± 53.4	< 0.001	118.1 ± 65.6	117.3 ± 48.3	0.503
**Triglyceride, mg/dL**	136.4 ± 106.4	169.5 ± 134.0	< 0.001	139.1 ± 109.3	144.1 ± 104.9	0.013

T2D, type 2 diabetes; OAG, open angle glaucoma; BP, blood pressure; BMI, body mass index; HDL, high-density lipoprotein; LDL, low-density lipoprotein.

**Fig 1:**
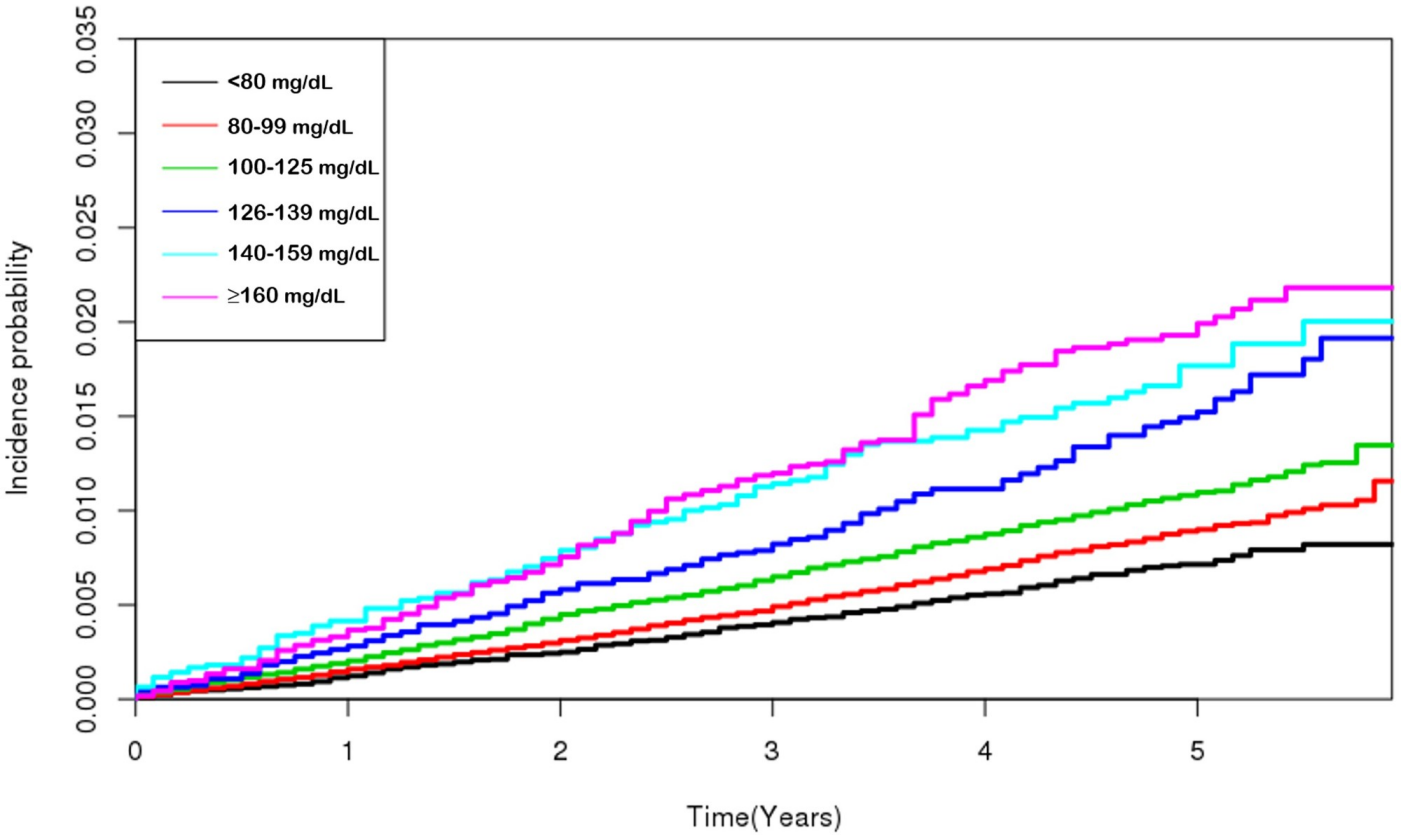
Cumulative incidence of open-angle glaucoma according to the fasting plasma glucose.

Table 2 shows that FPG was positively associated with the incidence of OAG. In an adjusted Cox proportional hazards model, subjects with the highest FPG level (≥ 160 mg/dL) had a higher HR [2.189; 95% confidence interval (CI): 1.779–2.695; *P* for trend < 0.001] for OAG compared with subjects with the lowest level (< 80 mg/dL) after adjustments for age and sex. This result persisted after further adjustments for the presence of T2D, current smoking, drinking, and exercise habits, diastolic BP, BMI, and total cholesterol level (HR: 1.588; 95% CI: 1.268–1.989; *P* for trend < 0.001). The cumulative incidence of OAG according to the FPG level is shown in Fig 1.

**Table 2:** Hazard ratios from multivariate Cox regression analysis for the development of primary open—Angle glaucoma according to fasting plasma glucose level in the cohort population.

Glucose (mg/dL)	N	OAG	IR	MODEL 1	*P* for trend	MODEL 2	*P* for trend
< 80	31,036	180	1.404	1 (ref.)	< 0.001	1 (ref.)	< 0.001
80–99	209,708	1488	1.734	1.193 (1.022, 1.393)		1.203 (1.030, 1.404)	
100–125	103,589	922	2.169	1.296 (1.104, 1.521)		1.244 (1.059, 1.462)	
126–139	11,067	135	2.995	1.543 (1.234, 1.930)		1.261 (1.000, 1.589)	
140–159	7,740	113	3.570	1.837 (1.451, 2.326)		1.387 (1.082, 1.777)	
≥ 160	11,236	178	3.968	2.189 (1.779, 2.695)		1.588 (1.268, 1.989)	

OAG, open angle glaucoma. Data are incidence rates (IR, per 1,000 person-years) adjusted for age and sex (model 1) and additionally adjusted for current smoking, drinking, and exercising habits, diastolic blood pressure, body mass index, total serum cholesterol level, and the presence of type 2 diabetes (model 2). Each fasting plasma glucose level indicates a comparison of the OAG risk between those with that FPG level or higher and those with a lower level.

Table 3 shows subgroup analyses according to age, BMI, and BP. Among subjects 40–64 years of age, those with the highest FPG level (≥ 160 mg/dL) had a higher HR (2.022; 95% CI: 1.494–2.736, *P* for trend < 0.001) compared with subjects with the lowest level (< 80 mg/dL) in model 2, whereas no trend was observed among subjects ≥ 65 years (HR: 1.047; 95% CI: 0.740, 1.482; *P* for trend = 0.560). Likewise, subjects with a BMI < 25 kg/m^2^ showed a stepwise increase in the rate of OAG development with increasing FPG level (HR; 1.817; 95% CI: 1.372–2.407; *P* for trend = 0.006), whereas no such trend was observed in subjects with a BMI > 25 kg/m^2^ (HR: 1.316; 95% CI: 0.896–1.931; *P* for trend = 0.280) in model 2. In addition, subgroup analyses based on BP indicated that subjects without hypertension showed a stepwise increase in the rate of OAG development with increasing FPG level (HR: 1.706; 95% CI: 1.180–2.467; *P* for trend < 0.001), whereas hypertensive subjects without and those with exposure to anti-hypertensive medications did not exhibit this trend in model 2 (HR: 1.202; 95% CI: 0.570–2.535; *P* for trend = 0.152 and HR: 1.454; 95% CI: 1.049–2.014; *P* for trend = 0.060, respectively).

**Table 3:** Multivariate Cox regression analyses of open-angle glaucoma according to fasting plasma glucose level in stratified subgroups based on age, body mass index, and presence of hypertension.

		Glucose (mg/dL)	N	OAG	IR (per 1,000)	MODEL 1	*P* for trend	MODEL 2	*P* for trend
**Age**	**40–64 years**	< 80	26,366	96	0.887	1 (ref.)	< 0.001	1 (ref.)	< 0.001
		80–99	176,653	906	1.265	1.359 (1.101, 1.677)		1.373 (1.112, 1.695)	
		100–125	81,609	494	1.485	1.408 (1.130, 1.753)		1.37 (1.098, 1.709)	
		126–139	7,877	71	2.212	1.839 (1.351, 2.502)		1.492 (1.083, 2.054)	
		140–159	5,534	58	2.571	2.073 (1.494, 2.877)		1.530 (1.081, 2.164)	
		≥ 160	8,501	116	3.430	2.928 (2.23, 3.843)		2.022 (1.494, 2.736)	
	**≥ 65 years**	< 80	4,670	84	4.215	1 (ref.)	0.009	1 (ref.)	0.555
		80–99	33,055	582	4.100	0.979 (0.779, 1.231)		0.988 (0.786, 1.243)	
		100–125	21,980	428	4.635	1.101 (0.871, 1.391)		1.058 (0.836, 1.338)	
		126–139	3,190	64	4.934	1.165 (0.842, 1.613)		0.987 (0.706, 1.38)	
		140–159	2,206	55	6.051	1.442 (1.026, 2.025)		1.150 (0.805, 1.641)	
		≥ 160	2,735	62	5.615	1.338 (0.964, 1.858)		1.047 (0.740, 1.482)	
**Body mass index**	**< 25 kg/m**^**2**^	< 80	23,146	128	1.345	1 (ref.)	< 0.001	1 (ref.)	< 0.001
		80–99	144,867	945	1.599	1.145 (0.952, 1.377)		1.153 (0.958, 1.387)	
		100–125	59,771	562	2.291	1.382 (1.140, 1.675)		1.333 (1.098, 1.618)	
		126–139	5,618	72	3.147	1.585 (1.186, 2.117)		1.295 (0.959, 1.749)	
		140–159	3,806	55	3.512	1.770 (1.289, 2.430)		1.339 (0.960, 1.868)	
		≥ 160	6,003	111	4.636	2.504 (1.940, 3.232)		1.817 (1.372, 2.407)	
	**≥ 25 kg/m**^**2**^	< 80	7,890	52	1.577	1 (ref.)	0.001	1 (ref.)	0.280
		80–99	64,841	543	2.031	1.271 (0.956, 1.690)		1.294 (0.974, 1.721)	
		100–125	43,818	360	2.003	1.145 (0.856, 1.531)		1.119 (0.835, 1.499)	
		126–139	5,449	63	2.840	1.438 (0.995, 2.077)		1.216 (0.834, 1.773)	
		140–159	3,934	58	3.628	1.825 (1.255, 2.656)		1.425 (0.963, 2.107)	
		≥ 160	5,233	67	3.203	1.747 (1.216, 2.510)		1.316 (0.896, 1.931)	
**Presence of Hypertension**	**Without hypertension**	< 80	23,154	96	1.014	1 (ref.)	< 0.001	1 (ref.)	< 0.001
		80–99	150,843	809	1.326	1.270 (1.028, 1.569)		1.277 (1.033, 1.579)	
		100–125	58,913	378	1.578	1.364 (1.090, 1.708)		1.346 (1.074, 1.688)	
		126–139	4,554	52	2.822	2.113 (1.506, 2.964)		1.805 (1.269, 2.569)	
		140–159	3,074	36	2.864	2.111 (1.438, 3.099)		1.674 (1.114, 2.515)	
		≥ 160	4,727	55	2.922	2.281 (1.637, 3.181)		1.706 (1.180, 2.467)	
	**Hypertension without prior exposure to anti-hypertensive medication**	< 80	2,231	20	2.147	1 (ref.)	0.130	1 (ref.)	0.152
		80–99	16,231	113	1.684	0.790 (0.491,1.272)		0.798 (0.496, 1.285)	
		100–125	11,259	207	45.423	1.125 (0.697, 1.815)		1.135 (0.702, 1.834)	
		126–139	1,197	9	1.876	0.842 (0.383, 1.850)		0.822 (0.370, 1.825)	
		140–159	675	4	1.484	0.710 (0.242, 2.079)		0.662 (0.221, 1.988)	
		≥ 160	1,214	13	2.696	1.285 (0.638, 2.587)		1.202 (0.570, 2.535)	
	**Hypertension with exposure to antihypertensive medication**	< 80	5,651	64	2.686	1 (ref.)	< 0.001	1 (ref.)	0.057
		80–99	42,634	566	3.130	1.175 (0.907, 1.522)		1.205 (0.931, 1.561)	
		100–125	33,417	437	3.120	1.138 (0.875, 1.48)		1.098 (0.844, 1.429)	
		126–139	5,316	74	3.388	1.205 (0.862, 1.684)		0.995 (0.707, 1.402)	
		140–159	3,991	73	4.455	1.604 (1.146, 2.243)		1.241 (0.876, 1.758)	
		≥ 160	5,295	110	5.185	1.960 (1.440, 2.668)		1.454 (1.049, 2.014)	

Data are incidence rates (IR, per 1,000 person-years) adjusted for age and sex (model 1) and additionally adjusted for current smoking, drinking, and exercising habits, diastolic blood pressure, body mass index, total serum cholesterol level, and the presence of type 2 diabetes (model 2). Each fasting plasma glucose level indicates a comparison of the OAG risk between those with that FPG level or higher and those with a lower level.

## Discussion

In this population-based cohort study of Korean adults ≥ 40 years of age, the FPG level was associated with an increased risk of OAG after adjusting for the presence of T2D, age, sex, smoking, drinking, and exercise habits, diastolic BP, BMI, and total cholesterol level. In addition, the association between FPG and the risk of OAG was specific to distinct subgroups: those 40–64 years of age, the nonobese subjects, and those without hypertension. Collectively, these findings suggest that the FPG level might be a risk factor for OAG development in the general population.

Glaucoma is characterized by the gradual loss of visual field corresponding to the loss of neural tissue in optic disc, and many of patients with glaucoma do not suffer visual symptom until the condition is at an advanced stage [6]. Therefore, it is not too much to emphasize the importance of health screening for glaucoma, because it enables early diagnosis and intervention to prevent glaucoma progression. However, screening with IOP measurement alone may not adequate to detect all cases of OAG. In addition, general screening with visual field examination is challenging, because the reliability of a single measurement may be low and several consistent measurements are needed to establish the presence of defects [26]. Therefore, targeted glaucoma screening of higher-risk groups may be the most cost-effective method [27]. In this cohort study, we found the FPG as a predictor of development of OAG in the general population. Our study suggests that the level of FPG can be utilized to select higher-risk groups to prevent OAG in healthy individual screening.

IOP is increased in patients with T2D [16, 28]. In several epidemiological studies, the relationship between T2D and OAG has been controversial [29–32]. However, a recent meta-analysis [17], which identified 47 studies including 2,981,342 individuals from 16 countries, showed that T2D and the duration of T2D were significantly associated with an increased risk of OAG.

Although the exact mechanism underlying this phenomenon is not known, several factors may have played a role. One reason for the association between the FPG level and OAG might be that the FPG level is positively correlated with the IOP [16–18, 28]. However, previous reports showed that the effect of the fasting glucose level on IOP elevation is somewhat limited [16, 17]. A meta-analyses showed that the pooled average increase in IOP associated with a 10 mg/dL increase in the FPG level was 0.09 mmHg [17]. Cohen et al [16], using data from 18,406 subjects, reported similar results, showing that for every 10 mg/dL increase in the FPG level, the IOP only increased by 0.09 mmHg in males and by 0.11 mmHg in females. Second, it is possible that a high FPG level may affect the retina and optic nerve head even before T2D onset. T2D and OAG share some pathophysiological mechanisms, such as endothelial dysfunction and impaired autoregulation [7, 33]. However, there have been reports that vascular endothelial dysfunction is present even during the prediabetic period due to increased insulin levels in plasma [34]. In the prediabetes stage, microvascular dysfunction is correlated with the insulin, rather than FPG level [12, 35]. In addition, functional changes and thinning of the inner retina due to neural degeneration have been reported, even before clinically visible retinal changes occur [8, 9]. The optic nerve head, the target site where glaucomatous damage occurs, is a mechanically vulnerable spot within an otherwise strong corneoscleral envelope [36]. Vessels and glial cells as well as the axons of retinal ganglion cells pass through the optic nerve head. This crowded structure can render the optic nerve head more vulnerable to vascular insults. Experimentally, a high FPG level itself exhibits a short-term protective effect during optic nerve stress [37]. However, decreased vessel density in the optic nerve head of hyperglycemic patients has been reported clinically [38]. In accordance with this, we also found that the incidence of T2D was increased with the baseline FPG level in subjects without T2DM, showing 1.2% of incidence in subjects with FPG < 80 mg/dl and 7.1% of incidence in those with FPG between 100 mg/dL and 126 mg/dL (S1 File). Increased incidence of T2DM in those with FPG level in the higher-normal range suggests that high FPG may be associated with increased risk of glaucoma in the context of prediabetes.

In this study, we used the FPG test, which is the preferred method of screening and diagnosis for diabetes. The FPG measures a person’s blood sugar level after fasting or not eating anything for at least 8 hours and is relatively less variable than HbA1c and a reliable method to detect hyperglycemia. In consistent with our results, Zhao et al [17] suggested the biomarkers of glucose metabolism such as the levels of fasting glucose, HbA1c and HOMA-IR in participants without diabetes increased the incidence of OAG in the general U.S. population. However, the Los Angeles Latino Eye Study, which is the cross sectional study in an adult Latino population, found the random glucose level ≥ 200 mg/dL did not show significant associations with the presence of OAG [19]. The different measurement method of the glucose metabolism may have affected the inconsistent results across the studies. In addition, considering the significant linear association of FPG and the incidence of OAG in our longitudinal study, it appears that the level of hyperglycemia is closely associated with the OAG.

Using subgroup analyses, the association of FPG with the incidence of OAG was especially strong in subjects 40–64 years of age and in nonobese and nonhypertensive subjects (Table 3). Older age is a risk factor not only for the diagnosis of glaucoma but also for its progression [6]. The apoptosis of retinal ganglion cells also increases with age [39]. In this regard, the differential association between FPG and OAG by age group may suggest that aging has a stronger influence on the development of OAG than the FPG level.

The relationship between BP and glaucoma has been inconsistent across the studies, because BP show nonlinear associations with OAG according to the level of BP, with low diastolic and high systolic BP being associated with OAG [21, 40, 41]. In accordance with former studies, our study showed that patients with OAG exhibited significantly higher systolic and diastolic BP, as well as higher proportion of hypertension, than those without OAG (Table 1). Interestingly, nonhypertensive subjects were at greater risk of developing OAG with increasing FPG level, while that trend was not observed in subjects with hypertension without prior exposure to antihypertensive medication (Table 3). A recent epidemiologic study using National Health and Nutrition Examination Survey data showed a U-shaped relationship between BP and the glaucoma in those without antihypertensive medications [40]. In this regard, our results may suggest that high systolic and diastolic BP have independent effect on the development of glaucoma, having a stronger influence than the FPG level.

We observed a stronger association between FPG and OAG in nonobese individuals. Inconsistent results on the association between BMI and glaucoma have been reported. Positive associations between IOP and BMI have been reported in both sexes among diverse ethnic groups [28, 42]. However, in a nationwide epidemiological study in the Republic of Korea, high fat mass was found to be protective against OAG [43]. Differential associations of metabolic syndrome components with OAG have also been reported according to obesity status, with positive associations of OAG with elevated BP, and high triglyceride level among the nonobese population, while no such associations were observed in the obese population [44]. The differential association of FPG with glaucoma by obesity is possibly associated with a higher prevalence of metabolic syndrome components such as hypertension in the obese population. In this regard, the high FPG seems to have greater effect on the development of glaucoma in nonobese population.

We found that subjects with T2DM exhibited significantly lower LDL, HDL, and total cholesterol level than subjects without T2DM, whereas TG level was significantly higher in subjects with T2DM (Table 1). The low total cholesterol and LDL cholesterol in subjects with T2DM may be associated with the higher prevalence of lipid-lowering medication users among individuals with T2DM. This is consistent with the most common pattern of dyslipidemia in patients with T2DM, in which patients shows elevated triglyceride levels and decreased HDL cholesterol levels [45].

The strengths of the present study are that it was a Korean population-based epidemiological cohort study involving a very large number of participants. The NHIS data have been validated by reference to the prevalence rates of 20 major diseases [21–23, 25, 46–48]. However, some limitations must be acknowledged. The most important limitation was the use of an insurance database. The diagnosis of OAG was defined based on ICD-10 codes, which may be inaccurate compared with diagnoses obtained from medical charts. To minimize inaccuracies, OAG was defined according to additional glaucoma medication use, rather than relying on the diagnostic code alone. In addition, there are possibility that some of the cases with neovascular glaucoma might be included as OAG. When we performed further analyses on the subjects who developed OAG, we found that only 5.5% of the subjects had undergone panretinal photocoagulation in an outpatient clinic or during vitrectomy, which may indicate that the subjects developed proliferative diabetic retinopathy. Considering that neovascular glaucoma is one of the end-stage complications of proliferative diabetic retinopathy, we considered the number of individuals with neovascular glaucoma might be negligible in our cohort. Second, claims data do not provide specific clinical examination data, such as IOP, visual field test results, or glucose tolerance indicators including postprandial glucose and HbA1c levels. Although claims data provide only information about a subject rather than precise clinical information, the use of an insurance database was valuable for evaluating the association between the FPG level and OAG incidence in this study. Third, HbA1c data were not available in this study and we used data based on a single FPG measurement, performed at clinical laboratories with standard quality assurance and control protocols in place. Information on FPG and other variables might vary in quality depending on the time and the hospital. However, a single FPG measurement obtained for clinical purposes was used as a diagnostic standard and matched the World Health Organization’s recommended approach for epidemiological studies [49].

In conclusion, this nationwide population-based cohort study showed that the FPG was associated with an increased risk of OAG. In addition, the relative risk of OAG associated with an increasing FPG level was greater at younger ages, in the nonobese population, and in subjects without hypertension. Our study results suggest that subjects with high FPG levels require special attention when screening for glaucoma.

## Supporting information

S1 Table Cumulative incidence of type 2 diabetes according to the fasting plasma glucose in subjects without type 2 diabetes. (DOCX)
