# Supplementary material for: Fasting plasma glucose level and the risk of open angle glaucoma: Nationwide population-based cohort study in Korea
Source: PLoS One. 2020 Sep 23;15(9):e0239529. doi: 10.1371/journal.pone.0239529 (PMC7510965; doi:10.1371/journal.pone.0239529)
Supplement: S1 Table — (DOCX) [file pone.0239529.s001.docx]

**S1 Table. Cumulative incidence of type 2 diabetes according to the fasting plasma glucose in subjects without type 2 diabetes**

|  |  | **Cumulative incidence of type 2 diabetes, n (%)** | | | | |
| --- | --- | --- | --- | --- | --- | --- |
| Baseline FPG  (mg/dL) | N | 1 year | 2 year | 3 year | 4 year | 5 year |
| < 80 | 30,180 | 36 (0.1) | 111 (0.4) | 191 (0.6) | 279 (0.9) | 367 (1.2) |
| 80 - 99 | 205,979 | 299 (0.1) | 1,021 (0.5) | 1,763 (0.9) | 2,519 (1.2) | 3,255 (1.6) |
| 100 - 125 | 94,183 | 770 (0.8) | 2,281 (2.4) | 3,921 (4.2) | 5,388 (5.7) | 6,733 (7.1) |
